# [Retracted] Calpastatin peptide attenuates early brain injury following experimental subarachnoid hemorrhage

**DOI:** 10.3892/etm.2024.12697

**Published:** 2024-08-28

**Authors:** Fei Teng, Yanxin Yin, Jia Guo, Ming Jiang

Exp Ther Med 19:2433–2440, 2020; DOI: 10.3892/etm.2020.8510

Following the publication of this paper, it was drawn to the Editor’s attention by a concerned reader that the “SAH+CP” image of the rat brain shown in Fig. 2A on p. 2436 was strikingly similar to an image (albeit with different dimensions) that appeared in another article published in the journal *Translational Stroke Research*, which was written by different authors at different research insititutes.

Owing to the fact that the contentious data in the above article has appeared in another publication, the Editor has decided that this paper should be retracted from the Journal. After having been in contact with the authors, they accepted the decision to retract the paper. The Editor apologizes to the readership for any inconvenience caused.

